# Integration of Digital Dental Casts in Cone-Beam Computed Tomography Scans

**DOI:** 10.5402/2012/949086

**Published:** 2012-09-23

**Authors:** Frits A. Rangel, Thomas J. J. Maal, Stefaan J. Bergé, Anne Marie Kuijpers-Jagtman

**Affiliations:** ^1^Department of Orthodontics and Craniofacial Biology, Radboud University Nijmegen Medical Centre, 309 Dentistry, P.O. Box 9101, 6500 HB Nijmegen, The Netherlands; ^2^Department of Oral and Craniomaxillofacial Surgery, Radboud University Nijmegen Medical Centre, Postal Number 590, P.O. Box 9101, 6500 HB Nijmegen, The Netherlands

## Abstract

Cone-beam computed tomography (CBCT) is widely used in maxillofacial surgery. The CBCT image of the dental arches, however, is of insufficient quality to use in digital planning of orthognathic surgery. Several authors have described methods to integrate digital dental casts into CBCT scans, but all reported methods have drawbacks. The aim of this feasibility study is to present a new simplified method to integrate digital dental casts into CBCT scans. In a patient scheduled for orthognathic surgery, titanium markers were glued to the gingiva. Next, a CBCT scan and dental impressions were made. During the impression-taking procedure, the titanium markers were transferred to the impression. The impressions were scanned, and all CBCT datasets were exported in DICOM format. The two datasets were matched, and the dentition derived from the scanned impressions was transferred to the CBCT of the patient. After matching the two datasets, the average distance between the corresponding markers was 0.1 mm. This novel method allows for the integration of digital dental casts into CBCT scans, overcoming problems such as unwanted extra radiation exposure, distortion of soft tissues due to the use of bite jigs, and time-consuming digital data handling.

## 1. Introduction

With the introduction of cone-beam computed tomography (CBCT), it became possible to obtain an accurate three-dimensional (3D) representation of the patient's head with much lower radiation exposure than multislice computed tomography (MSCT) and a much higher information content compared to two-dimensional (2D) radiographs. This has proven to be a useful tool in diagnosis and treatment planning for certain problems in the maxillofacial region [1, 2]. When these 3D diagnostic methods are combined with 3D planning software, orthognathic surgery can be planned digitally and then transferred to the patient, which may reduce errors in terms of materials, construction of appliances, and hand skills [3]. Using software programs, 3D reconstructions can be modified, and simulation of the proposed surgery can be performed.

Unfortunately, 3D virtual planning of orthognathic surgery still suffers from the disadvantages of CBCT imaging. For orthognathic surgery, a good representation of the dental surfaces and the occlusion is needed to properly position the dental arches. However, CBCT provides insufficient visualisation of the dental arches, since the teeth are not accurately rendered, and CBCT scans are subject to scattering from artefacts at the occlusal level [4–7]. 

Digital dental casts provide an accurate and reliable representation of the dentition [8]. Incorporating digital dental casts into the CBCT dataset provides a means of gaining adequate representation of the teeth in a CBCT scan. Since the introduction of 3D imaging devices, several procedures have been developed to integrate digital dental casts into 3D stereolithographic [9] and computerized models [5, 6, 10, 11]. Gateno et al. [5] developed a method to perform this fusion using a bite jig with fiducial markers attached to it. The patient wore the bite jig when the CBCT scan was made, and afterwards the bite jig was scanned together with the impressions. After data processing, the fiducial markers were visualized on both the CBCT scan and the scan of the bite jig with the impressions. With a surface-based rigid registration algorithm, both datasets were matched, using the fiducial markers as reference points. The disadvantage of this method is that the fiducial markers are positioned outside the mouth and produce distortion of the soft tissues; this prevents reliable judgement of the patient's soft tissue anatomy.

Swennen et al. [10] developed a triple scan method, using an impression tray in which both the upper and lower jaws are registered. For this procedure, a CBCT scan with a large field of view was made of the patient at rest. Next, a low-resolution CBCT scan with a small field of view was made, with the impression tray placed in the mouth. Finally, the impression tray was scanned separately with high-resolution. With a voxel-based rigid registration algorithm, the impression scan was placed into the CBCT scan of the patient, using the impression tray in the low-resolution scan as reference. The major disadvantage of this method is that (1) two CBCT scans of the patient are required, necessitating increased radiation exposure (2) the digital data handling processes are time consuming.

To overcome the above-mentioned problems, we developed a method in which titanium markers are placed on the gingiva. Using these markers, it is possible to match digital dental casts into CBCT data, without deformation of the soft tissues. The purpose of this study was to establish clinical feasibility of the 3D integration model of digital dental casts in CBCT scans.

## 2. Materials and Methods

**Figure 1:**
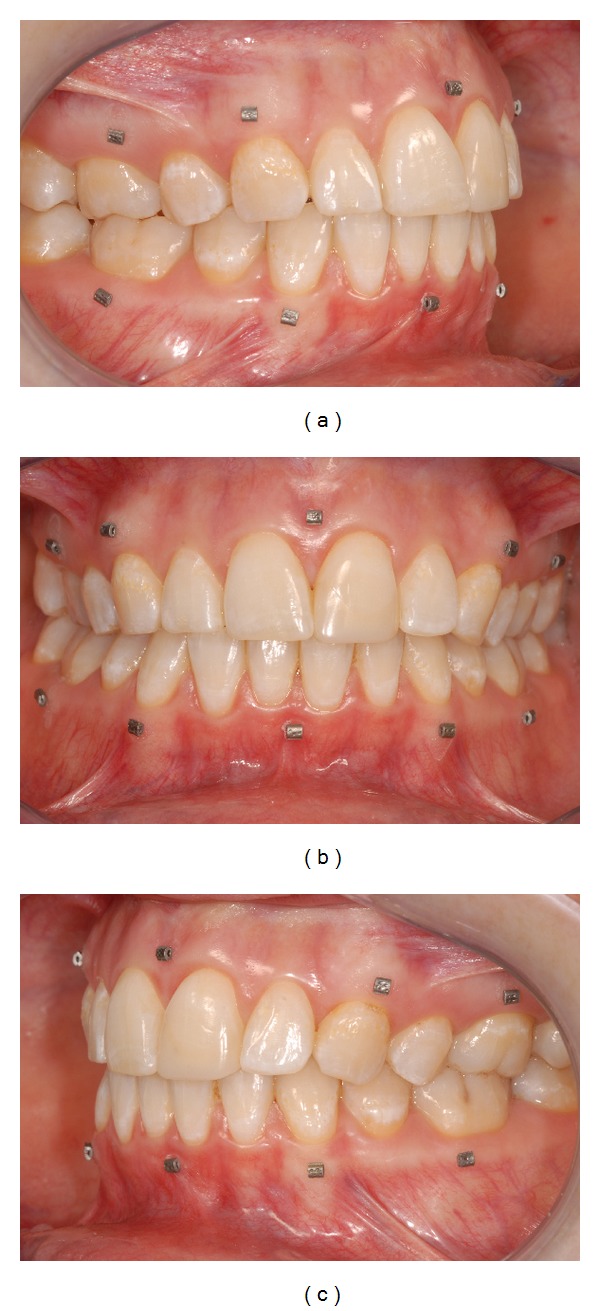
Example of titanium markers glued to the gingiva: (a) right view; (b) frontal view; (c) left view (different patient as in other pictures).

In one patient scheduled for orthognathic treatment, small rectangular (1 × 2 × 1.5 mm) titanium markers (speed split stops, Strite Industries Limited, Cambridge, ON, Canada) were glued to the gingiva using a N-butyl 2-cyanoacrylate tissue adhesive (Indermil, Henkel Ireland Ltd., Whitestown, Dublin, Ireland). The markers were placed, on the attached gingiva, 2 to 3 mm from the cervical margin, at the level of the midline, the canine, and the first molar. When all markers were placed the patient was left in the chair for five minutes to ensure that the adhesive dried completely.  Figure 1 shows an example of the placement of the markers in a different patient.

After the adhesive had dried, the patient was scanned using a standardized CBCT scanning protocol. CBCT scanning (I-CAT, Imaging Sciences International, Inc., Hatfield, USA) was performed in “extended height” mode (field of view: 17 cm diameter, 22 cm height; scan time 2 × 20 s; voxel size 0.4 mm) at 129 kV and 47.74 mA. When the scan was completed, impressions were taken using plastic impression trays (TP Orthodontics, Inc., La Porte, IN, USA) and orthodontic alginate (Cavex Orthotrace, Cavex Holland BV, Haarlem, The Netherlands). After hardening, the impressions were removed from the mouth with the markers embedded in the impression. Markers that remained attached to the gingiva were replaced in the marker spot in the impression. Some tissue adhesive remained on the gingiva of the patient. This was dissolved within an hour and did not give any discomfort to the patient. Finally, a wax bite was made (Tenastyle modelling wax, Kemdent Associated Dental Products Ltd, Wiltshire, UK) to place the models in the correct occlusion. To be approved, the wax bite should be completely bitten through, without any wax on the occluding points.

The impressions and the wax bite were sent to Orthoproof (Nieuwegein, The Netherlands), where the impressions and wax bite were scanned using a Flash CT scanner (model FCT-1600, Hytec Inc., Los Alamos, NM, USA) at 160 kV with a voxel resolution of 0.05 mm.

**Figure 2:**
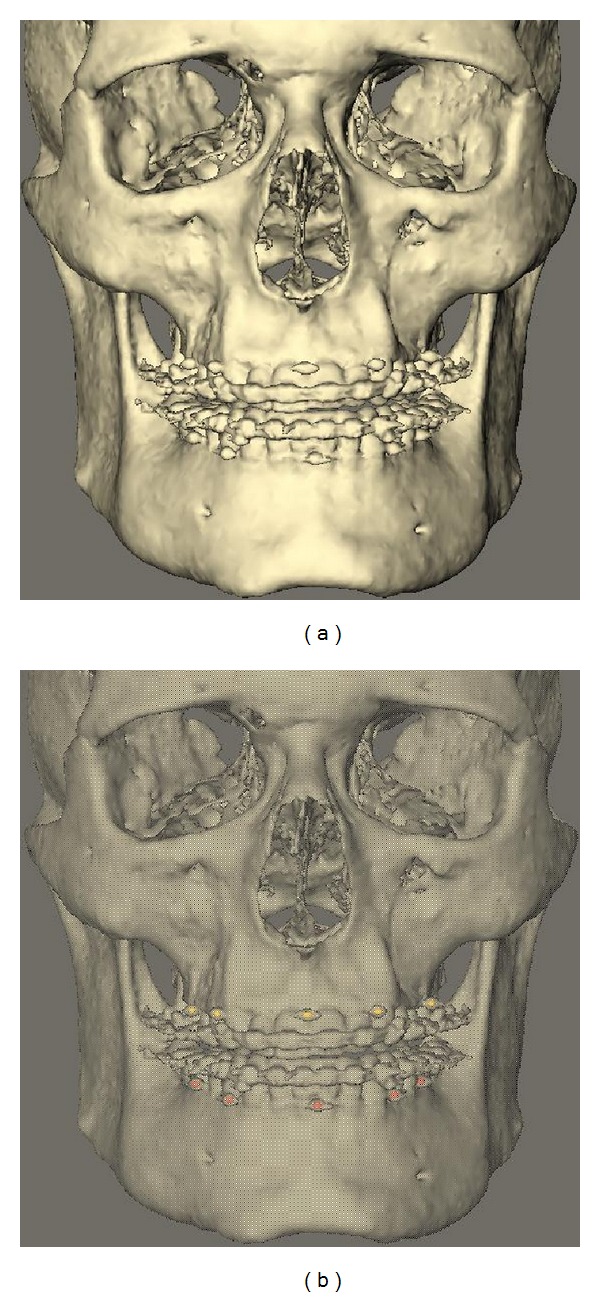
3D reconstruction patient scan: (a) normal reconstruction; (b) 3D reconstruction with markers extracted.

The scans of the patient's head and of the impressions were exported as DICOM (Digital Imaging and Communications in Medicine) datasets and imported into viewing software (Maxilim 2.3.0., Medicim NV, Mechelen, Belgium). From the scan of the patient's head, a 3D reconstruction was made of the bony structures. An isosurface was extracted by thresholding the DICOM images. For bony structures, a grey value of 276 was chosen as threshold value for the segmentation of the isosurfaces (Figure 2(a)). In a second step (in the same model, with the same reference frame), the markers were separately segmented, using a grey value of 3500 (Figure 2(b)). This 3D reconstruction will be called the 3D model.

**Figure 3:**
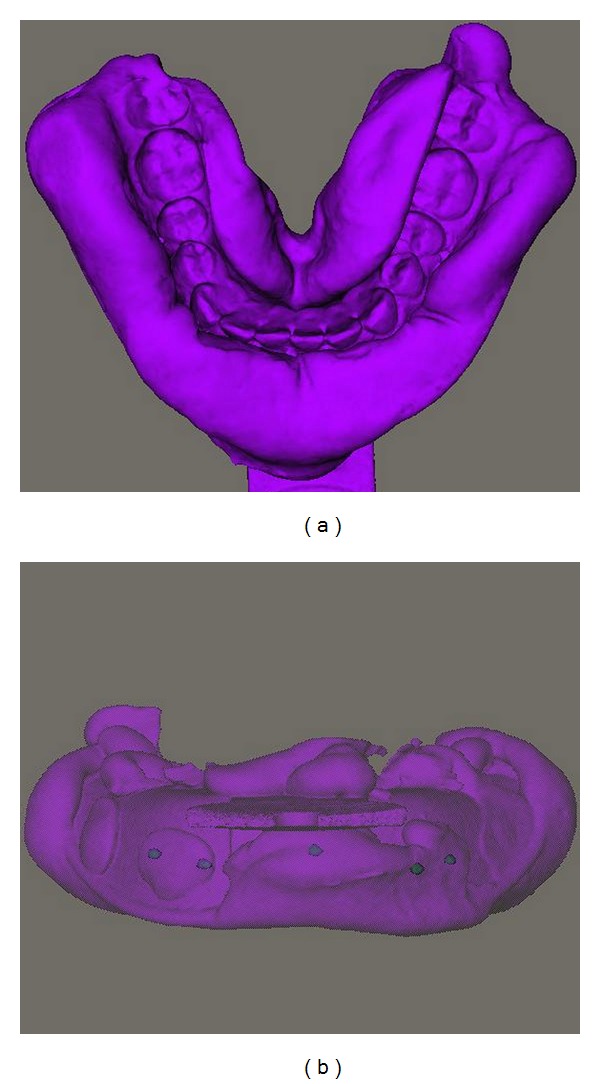
3D reconstruction impression scan: (a) normal reconstruction; (b) 3D reconstruction with markers extracted.

From the DICOM dataset of the impressions, a digital model was reconstructed using a grey value of −300 (Figure 3(a)). In a second step (in the same model, with the same reference frame), the markers were separately segmented, using a grey value of 3500 (Figure 3(b)). This 3D reconstruction will be called the digital impression.

After construction of the 3D model and the digital impression, the two models were fused in a six-step process.

**Figure 4:**
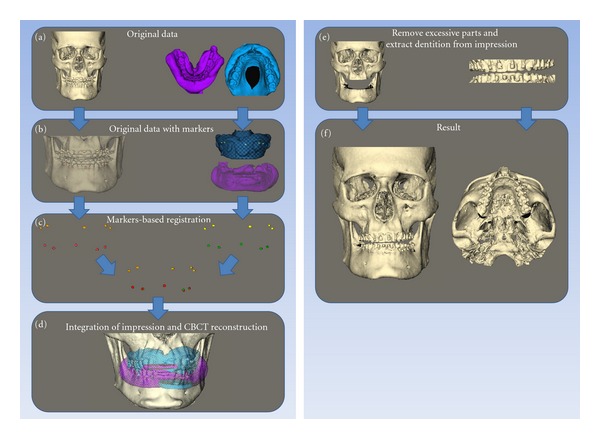
Matching procedure of the digital dental casts in the CBCT: (a) original datasets with 3D reconstruction patient scan and impression scan; (b) original datasets with markers extracted; (c) marker based registration; (d) integration of the reconstruction of the impression scans and CBCT reconstruction of the patients scan; (e) removal of excessive parts from the CBCT and extraction of dentition from the impressions; (f) final result.

- The DICOM data of both the patient's head and the impressions were imported into the Maxillim software (Figure 4(a)).
- Markers were extracted, in the same model, with the same reference frame (Figure 4(b)).
- The two models were matched on the titanium markers, using a Procrustes registration [12] (Figure 4(c)).
- Position of the impressions was checked after registration (Figure 4(d)).
- After registration of the markers, the impression was transformed to a negative image, resulting in visualization of the teeth. Next, the gingiva of the digital dental cast was removed, and the teeth were removed from the 3D model of the patient (Figure 4(e)).
- The 3D model and the digital dental cast were then fused and could be viewed in one screen (Figure 4(f)).

To evaluate the accuracy of the method, the distance between the corresponding markers of the 3D reconstruction and the digital impression was calculated after the matching procedure.

The time needed for the clinical procedure and data handling was recorded, using a digital stopwatch.

## 3. Results

The total time needed for placement of the markers in the mouth was approximately 10 minutes. Impression taking, including replacement of the markers into the impression, took approximately 5 minutes. The total time needed to produce the fusion model in the computer was approximately 15 minutes.

After registration of the markers, the distance between the markers of the 3D reconstruction and the digital impressions was calculated. The average distance between corresponding markers was 0.1 mm. The largest distance between two corresponding markers was 0.3 mm.

## 4. Discussion

This paper describes a method to fuse datasets of digital dental casts and CBCT scans using intraoral markers glued to the gingiva. This technique solves the problems associated with two different methods that are currently employed clinically [5, 10]. One uses an external set where fiducial markers are attached to a bite jig; the other requires that the patient undergoes two CBCT scans. The first technique is complicated by soft tissue deformation, the second by increased radiation exposure.

The main reason that we need the integration of tooth structures into CBCT scans is that metal artefacts produce scattering and noise, which makes it impossible to extract the teeth from CBCT scans. To get a proper matching, we need a matching area that is not influenced by scattering at the level of the occlusal plane or the brackets. In this study, we use the attached gingiva, 2-3 mm from the gingival margin, for the placement of the markers. This gives enough separation from the scatter region. The markers that we used were titanium markers, which showed some scattering after normal 3D reconstruction (with a grey value of 276). Due to this we extracted the markers separately, with a grey value of 3500. This resulted in extraction of only the titanium markers, without any scattering.

Using this new method, soft tissue deformations due to the use of bite jigs are avoided. The markers we used were small, so that no deformation of the soft tissue mask was created. Since the markers were also very light and glued to the attached gingiva, no distortion of the gingiva occurred. The tissue adhesive that was used in this study consisted of N-butyl 2-cyanoacrylate and was approved for clinical use in early 1996. Since then, it has been widely used for closure of superficial lacerations under low tension in a variety of different surgeries [13]. Our patient had good oral hygiene. It can be disputed whether markers can be attached to inflamed gingiva. However, since N-butyl 2-cyanoacrylate is used to close incisions instead of sutures [13, 14], where it is in close contact with body fluids, it seems reasonable to assume that gingival inflammation should not significantly affect adhesive strength. Further studies are required to determine the influence of gingival inflammation on the adhesive strength of N-butyl 2-cyanoacrylate.

The markers are attached firmly to the gingiva, but can come loose if the patient touches them with the tongue. Therefore, the patient was instructed not to do so. However, even if a marker did come loose, this would not be a major issue as we could get a very good registration of the dental casts on the CBCT when using only three markers per jaw.

It is possible that not all markers remain embedded in the alginate when the impression is removed. Even then, the location of the marker can be clearly identified in the alginate impression, and the rectangular markers can be accurately replaced in the impression. Markers that provide more mechanical retention in the alginate would solve this problem. However, such markers are not commercially available, and custom fabrication of titanium markers is quite expensive, which diminishes widespread practical use of this method. So far, replacement of markers into the impression was not a problem when matching the models, making custom markers unnecessary.

To combine different datasets into one 3D model, different matching techniques are available. Iterative closest point (ICP) surface matching is useful in matching two 3D surface reconstructions, for example, a 3D photograph and the soft tissue surface of a CBCT reconstruction. Maal et al. (2008) showed that the accuracy of this matching procedure is within 1.5 mm [15], which was considered acceptable for clinical use. For this integration model, surface matching is not applicable. For a proper surface matching procedure, usually more than 10.000 corresponding points are used to get a reliable result. As explained above, there are scattering artefacts from the brackets at the occlusal level and hence a large difference in the surface structure at the occlusal level of the two datasets. Therefore, we used titanium markers, which need a different matching procedure.

For matching CT scans and MRI scans, often voxel based registrations are used [16]. A voxel based registration uses the grey values to detect corresponding patterns in both scans. When impressions are scanned to produce a digital dental cast, the negative of the shape is used to create the dentition. This gives only one grey value for the scanned impression. In the CBCT scan of the patient, soft tissues and hard tissues give a pattern of grey values. Since there is no pattern of grey values in the scanned impression, it is not possible to match these two datasets.

In our study, a Procrustes matching method was used to align both datasets [12]. Using Maxilim software, we were able to extract the markers out of the DICOM datasets, using a grey value of 3500. By choosing a high grey value of 3500, we ensured that only the real marker value was used, and noise was disregarded. Choosing a grey value of 3500 also affects the reconstruction of the correct dimensions of the marker. The segmentation procedure will exclude outlying voxels at the edges, ensuring a round digital marker. However, for matching of the markers, the middle point of each marker was used. Thus, any loss due to segmentation will occur on all sides of the marker, and would not affect the middle point.

After segmentation, the markers of both datasets were aligned using the Procrustes method to a best fit of the five markers. The corresponding markers are matched as a best fit, without any scaling (i.e., only by rotation and translation). To visualize the accuracy of the matching procedure, an average distance between the markers was calculated. The average distance was 0.1 mm. The largest distance between two corresponding markers in this study was 0.3 mm.

**Figure 5:**
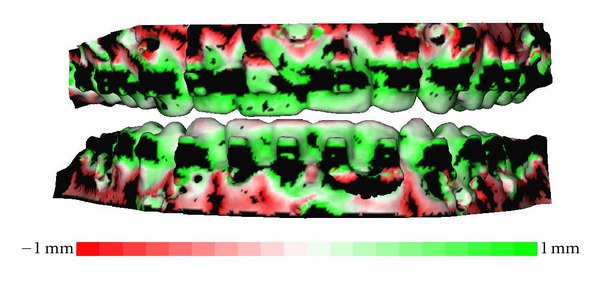
Distance map of the matched dentition of the CBCT of the patient and the impressions. It ranges from −1.0 mm to 1.0 mm.

One could argue that deformation of the impression material during removal of the impression would lead to poor matching of the corresponding markers. However, the average distance between the markers was found to be very small, so the matching of the markers appeared to be good. The distance map of the matched dentition, of the 3D model and the digital impression also shows very little variation between the two surfaces (Figure 5). All incisal and occlusal surfaces are coloured light green or light red, close to white, which means only a 0.1-0.2 mm difference between the two surfaces. Around the brackets, the distance kit is coloured black, indicating that the distance between the two surfaces is more than 1 mm. This is due to the scattering artefacts produced by the metal brackets. The combination of these two measurements shows that deformation of the impression material is negligible.

As described earlier, there are other methods to fuse digital datasets of the dentition and the skull [10]. The advantage of the method presented here is reduced radiation exposure, since only one CBCT of the patient is needed. A second advantage is more rapid processing of the digital data handling processes. Using the proposed new method, the extra time needed for marker placement is approximately 10 minutes. Additionally, time is needed for data processing, until the fusion model is complete; this takes approximately 15 minutes. Using the previously proposed method of Swennen et al. [10], the data handling processes take about 50 minutes. 

In conclusion, the proposed method is a promising approach for the integration of digital models into CBCT scans. Compared to other previously described methods, radiation exposure for the patient is lower, the soft tissues are not distorted, and digital data handling processes are faster. A larger patient sample is needed to validate this new method.
